# Supplementary material for: Dynamic light filtering over dermal opsin as a sensory feedback system in fish color change
Source: Nat Commun. 2023 Aug 22;14:4642. doi: 10.1038/s41467-023-40166-4 (PMC10444757; doi:10.1038/s41467-023-40166-4)
Supplement: Supplementary file 1 — Supplementary Information [file 41467_2023_40166_MOESM1_ESM.pdf]

Electronic Supplementary Materials for

Dynamic Light Filtering over Dermal Opsin as a Sensory Feedback System in Fish Color Change

Lorian E. Schweikert, Laura E. Bagge, Lydia F. Naughton, Jacob R. Bolin, Benjamin R. Wheeler, Michael S. Grace, Heather D. Bracken-Grissom, and Sönke Johnsen

Supporting Information:

Supplementary Figure 1

Supplementary Figure 2

Supplementary Figure 3

Supplemental Figures

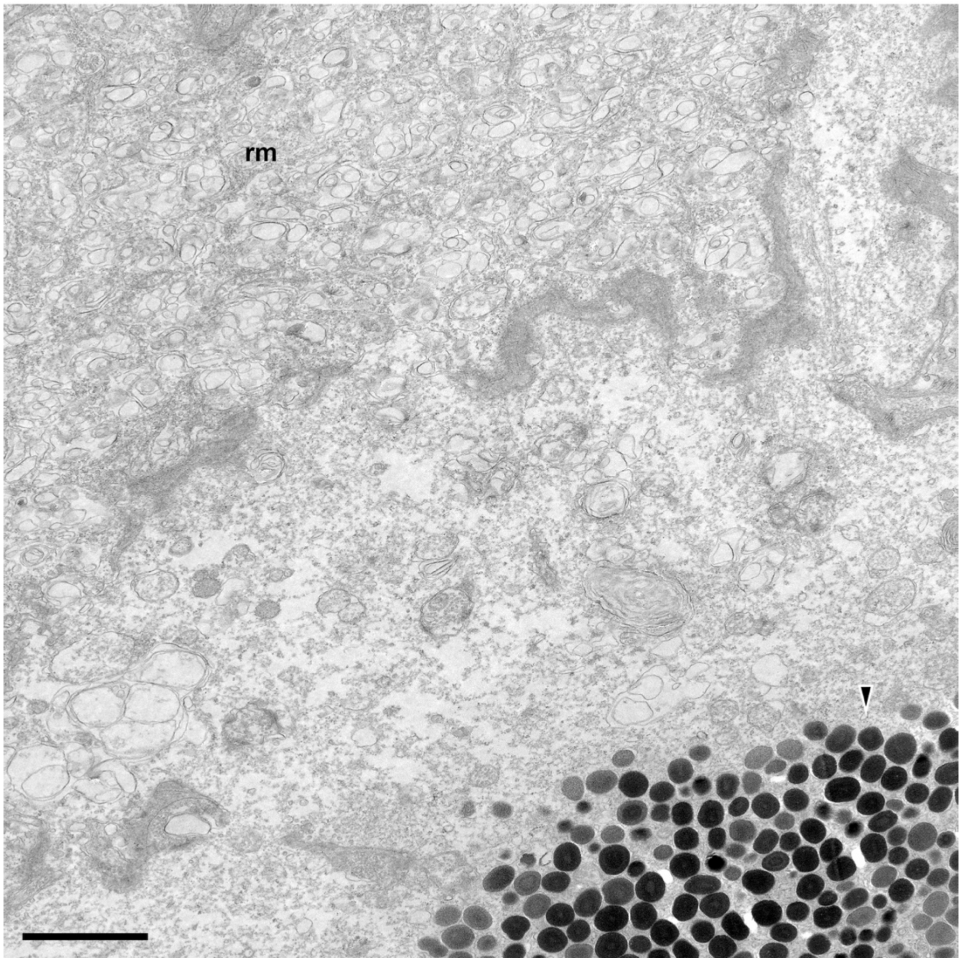

Supplementary Figure 1: An en face transmission electron micrograph of a melanophore and underlying membrane-filled cell. The section was cut tangential to the surface of the skin, exposing a portion of a melanophore (open triangle) and its underlying cell. The reticulated membrane structure (rm) has a morphology that is uniform in appearance when it is viewed horizontally (as shown here) or in cross section (e.g., Fig. S2). Scale bar equals 2 μm.

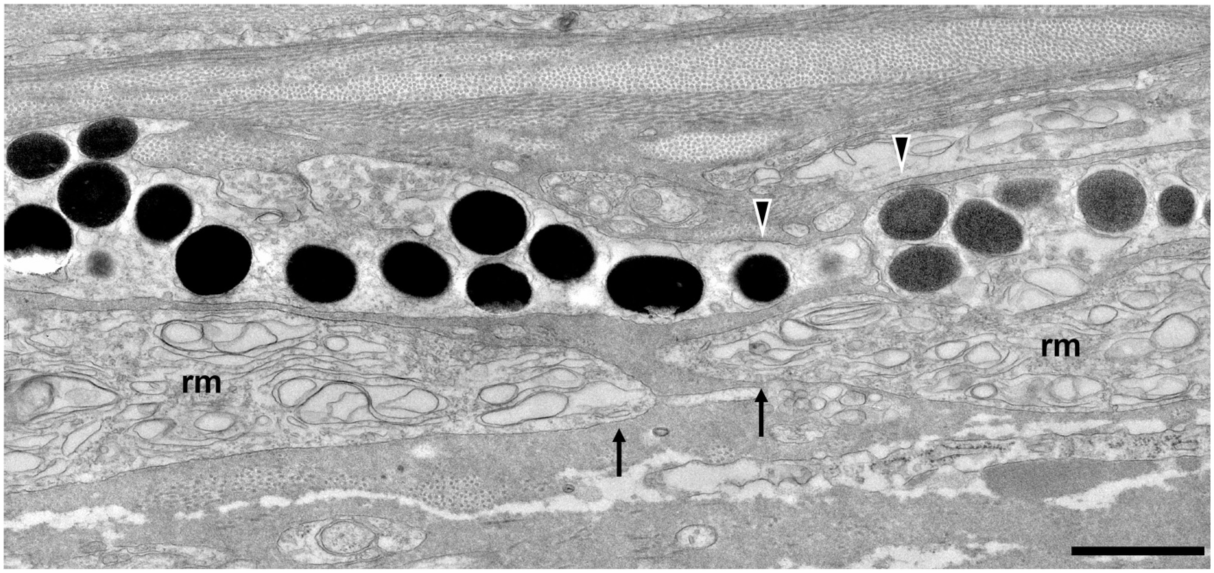

Supplementary Figure 2: A discrete population of morphologically-specialized cells are found under contiguous chromatophores. Cells filled with reticulated membrane are beneath and aligned with chromatophores. The margins of the two melanophores are indicated by the black triangles and those of the underlying cells are indicated by the black arrows. rm = reticulated membrane. Scale bar equals 1 μm.

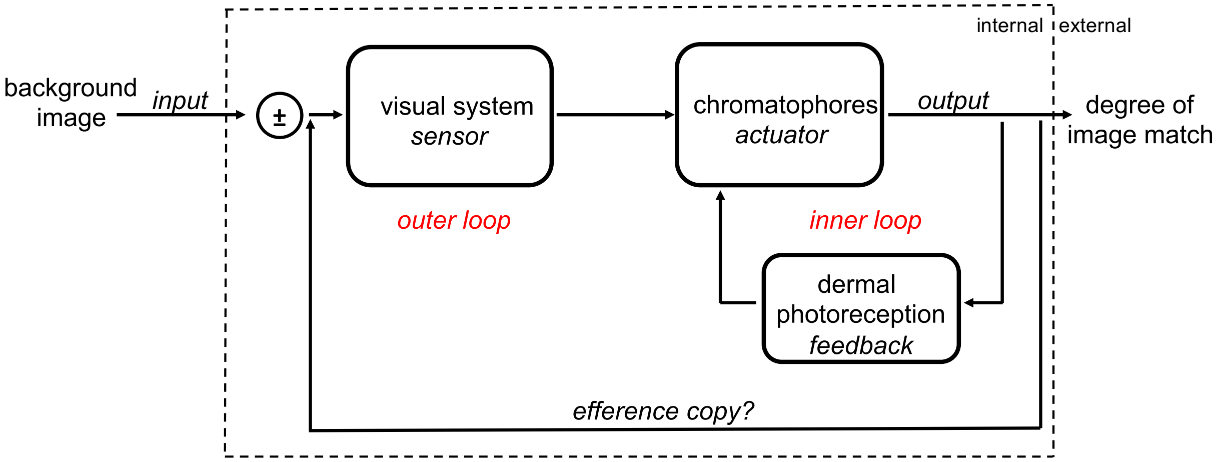

Supplementary Figure 3: A simplified block diagram of the sensory feedback model for the control of background-matching color change. The reference signal is the background image. The difference between the background image and the degree of image match (the error signal) is assessed by the outer loop. Assessment of the color change output by dermal photoreceptors permits feedback information to be sent to chromatophores via the inner loop. Together, the two control loops may work together to produce color change outputs at the system level.
