# Supplementary material for: Dynamic light filtering over dermal opsin as a sensory feedback system in fish color change
Source: Nat Commun. 2023 Aug 22;14:4642. doi: 10.1038/s41467-023-40166-4 (PMC10444757; doi:10.1038/s41467-023-40166-4)
Supplement: Supplementary file 3 — Reporting Summary [file 41467_2023_40166_MOESM3_ESM.pdf]

nature portfolio

Corresponding author(s): Lorian E. Schweikert

Last updated by author(s): Jul 5, 2023

Reporting Summary

Nature Portfolio wishes to improve the reproducibility of the work that we publish. This form provides structure for consistency and transparency in reporting. For further information on Nature Portfolio policies, see our Editorial Policies and the Editorial Policy Checklist.

Statistics

For all statistical analyses, confirm that the following items are present in the figure legend, table legend, main text, or Methods section.

n/a	Confirmed	
☐	☒	The exact sample size (*n*) for each experimental group/condition, given as a discrete number and unit of measurement
☒	☐	A statement on whether measurements were taken from distinct samples or whether the same sample was measured repeatedly
☒	☐	The statistical test(s) used AND whether they are one- or two-sided<br>*Only common tests should be described solely by name; describe more complex techniques in the Methods section.*
☒	☐	A description of all covariates tested
☒	☐	A description of any assumptions or corrections, such as tests of normality and adjustment for multiple comparisons
☐	☒	A full description of the statistical parameters including central tendency (e.g. means) or other basic estimates (e.g. regression coefficient) AND variation (e.g. standard deviation) or associated estimates of uncertainty (e.g. confidence intervals)
☒	☐	For null hypothesis testing, the test statistic (e.g. *F*, *t*, *r*) with confidence intervals, effect sizes, degrees of freedom and *P* value noted<br>*Give P values as exact values whenever suitable.*
☒	☐	For Bayesian analysis, information on the choice of priors and Markov chain Monte Carlo settings
☒	☐	For hierarchical and complex designs, identification of the appropriate level for tests and full reporting of outcomes
☒	☐	Estimates of effect sizes (e.g. Cohen's *d*, Pearson's *r*), indicating how they were calculated

Our web collection on statistics for biologists contains articles on many of the points above.

Software and code

Policy information about availability of computer code

Data collection	No code was used to collect the data for this study. However, the software used to collect data was 1) National Center for Biotechnology Information BLASTx (Basic Local Alignment search Tool; v2.13.0) for sequence identification, 2) CLC Viewer Software Version 8.0 (Qiagen, Redwood City, CA) for sequence alignment and 3) OceanView Software (v1.6.7; Ocean Optics Inc., Dunedin, FL) for microspectrophotometry.
Data analysis	No code was used to analyze the data. However, Microsoft Excel software (v16.70). Also, Adobe Photoshop (v24.0) were used in preparing the manuscript.

For manuscripts utilizing custom algorithms or software that are central to the research but not yet described in published literature, software must be made available to editors and reviewers. We strongly encourage code deposition in a community repository (e.g. GitHub). See the Nature Portfolio guidelines for submitting code & software for further information.

Data

Policy information about availability of data

All manuscripts must include a data availability statement. This statement should provide the following information, where applicable:

- Accession codes, unique identifiers, or web links for publicly available datasets
- A description of any restrictions on data availability
- For clinical datasets or third party data, please ensure that the statement adheres to our policy

As part of this study, we examined the SWS1 opsin gene sequence of Aulonocara hueseri (Genbank accession AY775100.1; https://www.ncbi.nlm.nih.gov/nuccore/AY775100.1/) and aligned it to the SWS1 opsin sequence of Lachnolaimus maximus (Genbank Accession: PRJNA386691; https://www.ncbi.nlm.nih.gov/bioproject/

PRJNA386691). All other data included in this study are available in the main text and supplemental materials.

Research involving human participants, their data, or biological material

Policy information about studies with human participants or human data. See also policy information about sex, gender (identity/presentation), and sexual orientation and race, ethnicity and racism.

Reporting on sex and gender	NA
Reporting on race, ethnicity, or other socially relevant groupings	NA
Population characteristics	NA
Recruitment	NA
Ethics oversight	NA

Note that full information on the approval of the study protocol must also be provided in the manuscript.

Field-specific reporting

Please select the one below that is the best fit for your research. If you are not sure, read the appropriate sections before making your selection.

[x] Life sciences [ ] Behavioural & social sciences [ ] Ecological, evolutionary & environmental sciences

For a reference copy of the document with all sections, see nature.com/documents/nr-reporting-summary-flat.pdf

Life sciences study design

All studies must disclose on these points even when the disclosure is negative.

Sample size	The study specimens were hogfish (Lachnolaimus maximus; family Labridae) ranging in total length from 16.5 to 35.5 cm (N = 16 fish, total). Hogfish are a protogynous hermaphroditic reef fish, switching from female to male as required at roughly 30.5 cm fork length. Thus, the specimens included in this study are primarily female, representing subadult to adult life-history stages. All of the data presented in the study is qualitative (e.g., expression presence and absence, micrograph assessment, gene sequences and spectrophotometry characterizations). Our sample sizes were determined using a precedent in the literature set by Taylor and colleagues 2011. They completed a similar characterization of photoreceptive tissue (i.e., retina) using the same methods in our study: light microscopy, immunofluorescence, transmission electron microscopy, and microspectrophotometry. Our sample sizes met or exceeded those listed in this previous work for each method.<br><br>Taylor, S., Loew, E. , Grace, M. Developmental shifts in functional morphology of the retina in Atlantic tarpon, Megalops atlanticus (Elopomorpha: Teleostei) between four ecologically distinct life-history stages. Vis. neurosci. 28, 309-323.(2011).
Data exclusions	No data were excluded from the analyses.
Replication	Experiments associated with each method were replicated multiple times independently. For light microscopy, micrographs were collected across six independent sample preparations across three fish. For immunofluorescence, experiments were run three times independently on separate days at the Florida Institute of Technology and replicated an additional two times, at Duke University. The TEM and immunogold images were collected from two separate sample preparations, which had been analyzed at UCSD and UNCW, respectively. For microspectrophotometry, data were collected across three fish that had been sampled over separate days. Spectra for n=60 cells/ chromatophore type were collected using no less than 10 scales sampled from each fish body.
Randomization	Skin samples were selected at random (haphazardly) from different body regions (e.g., dorsal, ventral, and caudal body regions) for analysis, often selected by more than one investigator for a given analysis, independently.
Blinding	Blinding was not relevant to this study because there was no statistical comparison of groups.

Reporting for specific materials, systems and methods

We require information from authors about some types of materials, experimental systems and methods used in many studies. Here, indicate whether each material, system or method listed is relevant to your study. If you are not sure if a list item applies to your research, read the appropriate section before selecting a response.

Materials & experimental systems

n/a	Involved in the study	
[ ]	[x]	Antibodies
[x]	[ ]	Eukaryotic cell lines
[x]	[ ]	Palaeontology and archaeology
[ ]	[x]	Animals and other organisms
[x]	[ ]	Clinical data
[x]	[ ]	Dual use research of concern
[x]	[ ]	Plants

Methods

n/a	Involved in the study	
[x]	[ ]	ChIP-seq
[x]	[ ]	Flow cytometry
[x]	[ ]	MRI-based neuroimaging

Antibodies

Antibodies used

All primary and secondary antisera were commercially-obtained. The primary antiserum was an SWS1 opsin antiserum was raised against a recombinant human SWS1 immunogen (1:200-1000 concentration, polyclonal, EMD Millipore catalog# AB5407). We also used rod opsin (rhodopsin; RH1) antiserum (1:500 concentration, monoclonal, EMD Millipore catalog# MAB5316)to counterstain rod photoreceptor outer segments in the hogfish retina. The secondary antisera were conjugated to Alexa Fluor fluorescent dyes for immunofluorescence (1:500, Thermofisher Scientific catalog # A-11008 and A-21422) and to 25-nm colloidal gold in a BSA solution for immunogold labeling (1:40, Electron Microscopy Sciences, Hatfield, PA).

Validation

The SWS1 opsin and rhodopsin antisera used here have known specificity and cross reactivity to SWS1 opsins in diverse species. Specificity and sensitivity of all antisera were validated by the lack of expression in control preparations and positive labeling of a rod cone and rod photoreceptor populations in cross section of hogfish retina.

Animals and other research organisms

Policy information about studies involving animals; ARRIVE guidelines recommended for reporting animal research, and Sex and Gender in Research

Laboratory animals

NA

Wild animals

The study specimens were hogfish (Lachnolaimus maximus; family Labridae) ranging in total length from 16.5 to 35.5 cm (N = 16 fish, total). Wild-caught hogfish were collected under a Florida Fish and Wildlife Conservation Commission special activity license (SAL-16-1822A-SR), by the approval of the Institutional Animal Care and Use Committees at Duke University (protocol #A233-16-10), Florida International University (protocol #IACUC-19-024), and the University of North Carolina Wilmington (protocol # A2020-016). Commercially-obtained hogfish were purchased from Dynasty Marine Associates (Marathon, FL) and Gulf Specimen Marine Laboratories, Inc. (Panacea, FL). All animals were humanely euthanized by either overdose of MS-222 (Tricaine) or eugenol (clove oil) according to approved IACUC procedures. For microspectrophotometry only, fresh carcasses provided tissues of adequate quality, which were obtained from recreational fisherman via Wrightsville Beach Diving Spearfishing Charter (Wrightsville Beach, NC). All hogfish used, both wild-caught by investigators and those commercially obtained, were captured from coastal Florida, along the eastern Gulf of Mexico.

All animals obtained for this study were euthanized for the subsequent histological analyses reported here. Wild-caught animals were captured by hook-and-line, maintained in large aerated coolers, and transported by car to the laboratory. According to the 'Guidelines for the Care and Use of Fish in Research' (Jenkins et al., 2014), euthanasia was accomplished using eugenol at a concentration of 400mg/L of seawater or MS-222 (tricaine) at a concentration of 10g/L. Individual fish were transfered to a covered 5-gallon bucket of seawater taken from the animal's home tank containing the dissolved agent. Euthanasia was accomplished when gill ventilation ceased for a period of at least two minutes prior to surgical dislocation of the spine.

Jenkins, J. A., Bart Jr, H. L., Bowker, J. D., Bowser, P. R., MacMillan, J. R., Nickum, J. G., ... & Warkentine, B. E. Guidelines for the Use of Fishes in Research. Bethesda, Maryland, USA: American Fisheries Society. (2014).

Reporting on sex

Hogfish are a protogynous hermaphroditic reef fish, switching from female to male as required at roughly 30.5 cm fork length 33. Thus, the specimens included in this study are primarily female, representing subadult to adult life-history stages.

Field-collected samples

For laboratory aquaria, seawater was mixed using Instant Ocean Sea Salt (Blacksburg, VA) and maintained to match natural habitat conditions of salinity (~32 ppt), temperature (~24 degC), and pH (pH = 8.0). Upon arrival, fish were acclimated to laboratory seawater, then transferred to home aquaria using a soft-sided net. Fish were housed together across 20- to 50- gallon tanks with recirculating filtration, and held under broad-spectrum aquarium lighting within a 12:12 hr daily light:dark cycle. The number of fish held at one time within these aquaria was size dependent, with no more than one fish held per 10 gallons of seawater. Fish were provided enrichment through the placement of large PVC pipe pieces in home tanks in which the fish could swim. Water quality was tested daily and water changes occurred as needed, with no less than one water change per week. Animals were fed pieces of food-grade frozen shrimp ad libitum. Animals were held in captivity for a period no longer than 4 weeks prior to euthanasia for the planned histological experiments.

Ethics oversight

Hogfish were collected under the approval of a Florida Fish and Wildlife Conservation Commission special activity license (SAL-16-1822A-SR), the Institutional Animal Care and Use Committees at Duke University (protocol #A233-16-10), Florida International University (protocol #IACUC-19-024), and the University of North Carolina Wilmington (protocol # A2020-016).

Note that full information on the approval of the study protocol must also be provided in the manuscript.
